# An Analysis of Anthropometric Indicators and Modifiable Lifestyle Parameters Associated with Hypertensive Nephropathy

**DOI:** 10.1155/2016/6598921

**Published:** 2016-09-27

**Authors:** Christiana Aryee, William K. B. A. Owiredu, James Osei-Yeboah, Ellis Owusu-Dabo, Edwin F. Laing, Isaac K. Owusu

**Affiliations:** ^1^Department of Molecular Medicine, School of Medical Sciences, Kwame Nkrumah University of Science and Technology, Kumasi, Ghana; ^2^Becton Dickinson Biosciences Technical Services, West Africa, Accra, Ghana; ^3^Department of Clinical Biochemistry, Diagnostic Directorate, Komfo Anokye Teaching Hospital, Kumasi, Ghana; ^4^Department of Medical Laboratory Sciences, School of Allied Health Sciences, University of Health and Allied Sciences, Ho, Ghana; ^5^School of Public Health, Kwame Nkrumah University of Science and Technology, Kumasi, Ghana; ^6^Kumasi Centre for Collaborative Research in Tropical Medicine, Kwame Nkrumah University of Science and Technology, Kumasi, Ghana; ^7^Department of Medicine, School of Medical Sciences, Kwame Nkrumah University of Science and Technology and Komfo Anokye Teaching Hospital, Kumasi, Ghana

## Abstract

The surge in prevalence of chronic noncommunicable diseases like hypertension and chronic kidney disease has been linked with modifiable lifestyle practices and increased body fat. This study sought to compare the association between different modifiable lifestyle practices, adiposity indices, renal function parameters, and hypertension as well as the predictive implications for levels of these parameters in target cardiac organ damage among an urban Ghanaian hypertensive population. Using a hospital-based case-control study design, 241 Ghanaian indigenes from the Kumasi metropolis were recruited for this study. The case group was made up of 180 hypertensives and 61 normotensives served as controls. In addition to sociodemographic data, standard haemodynamic, anthropometric, renal function, and cardiac organ damage assessments were done. The prevalence of chronic kidney disease (CKD) ranged from 13.3% to 16.6% depending on the equation used in estimating the glomerular filtration rate (eGFR). Percentage cluster distribution by chronic kidney disease was observed to be significantly tilted toward the upper quartiles (3rd and 4th) of the haemodynamic parameters measured. Chronic kidney disease was significantly higher among self-reported smokers and alcoholic hypertensives. In this urban population, adiposity was associated with hypertension and renal insufficiency. Chronic kidney disease was associated with hypertension and cardiac abnormalities.

## 1. Background

High blood pressure is a major health problem all over the world [1]. Ghana and other countries in Sub-Saharan Africa (SSA) are experiencing an epidemic of cardiovascular diseases propelled by rapidly increasing rates of hypertension [2]. A reciprocal or bidirectional relationship has been postulated for hypertension and end-stage chronic kidney disease (ESCKD) [3]. Hypertension is a key pathogenic factor linked to deterioration of kidney function, whilst the most common forms of secondary hypertension are attributable to chronic kidney disease [4–6]. According to Commodore-Mensah et al. [7], hypertension is the leading cause of renal failure in Ghana, whilst Owiredu et al. [8] estimated a 20-fold higher increased risk of death through cardiovascular complications among chronic kidney disease (CKD) patients than any other cause in the general population.

The upsurge in chronic noncommunicable diseases has been attributed to modifiable lifestyle practices characterized by sedentary lifestyle and a resultant obesity [9–13]. Evidence that hypertension is related to increases in body fat is well established in the literature [14–22]. Epidemiological studies of the impact of obesity on outcome in chronic kidney disease (CKD) remain conflicting, with several well-designed studies even suggesting a survival advantage for obese end-stage renal disease (ESRD) patients [23–25].

This study sought to compare the association between different modifiable lifestyle practices, adiposity indices, renal function parameters, and hypertension as well as the predictive implications for levels of these parameters in target cardiac organ damage among an urban Ghanaian hypertensive population.

## 2. Material and Methods

### 2.1. Study Participants

A hospital-based case-control study was conducted between November 2012 and September 2013. Two hundred and forty-one participants were involved in this study. One hundred and eighty (108) nondiabetic hypertensive patients were attending clinic at the Komfo Anokye Teaching Hospital (KATH) and the Precise Specialist Clinic, all in Kumasi, Ghana, and sixty-one (61) age-matched normotensive controls were from the Kumasi metropolis. The study participants were recruited from a population of adult individuals between the ages of 22 and 87 years. Criteria for the case group were patients diagnosed with hypertension who were not suffering from diabetes and were of consent age. The control group were normotensive age-matched healthy individuals with no past history of diabetes, cardiac, renal, and hepatic dysfunction, or dyslipidaemia, living in the Kumasi metropolis, who consented to participate in this study. The detailed characteristics of study participants have been published in an earlier work [26].

### 2.2. Sociodemographic Data Capture (Questionnaire)

Self-reported structured questionnaire was administered to determine duration of hypertension and treatment status, smoking status, alcohol intake, educational level, physical activity levels, occupation, the usage of nonprescribed orthodox and herbal medications, family history of hypertension, current and past symptoms of cerebrovascular disease, and peripheral vascular and coronary heart diseases.

### 2.3. Blood Pressure (BP) Measurement

Blood pressure (BP) and pulse rate measurements were done using the Omron M5-I digital fully automatic blood pressure monitor (OMRON Healthcare Europe BV Wegalaan 57 NL-2132 JD Hoofddorp). After participants had sat quietly for at least ten minutes, three measurements were taken at one-minute intervals on the right arm in a seated position, with arm supported at heart level and feet flat on the floor using an appropriate sized cuff. Hypertension was diagnosed when the mean of the second and third blood pressure (BP) measurements was equal or above 140/90 mmHg or when participants reported use of antihypertensive medication which was verified from their hospital files [27, 28].

### 2.4. Anthropometric Variables

Anthropometric measurements included height to the nearest millimeter without shoes and weight to the nearest 0.1 kg in light clothing. Subjects were weighed on a bathroom scale (Zhongshan Camry Electronic Co. Ltd., Guangdong, China) and their height was measured with a Seca stadiometer with the participant standing erect with back straight, heels together, and feet slightly apart at a 60-degree angle. Waist circumference (to the nearest centimeter) was measured with a Gulick II spring-loaded measuring tape (Gay Mills, WI) midway between the inferior angles of the ribs and the suprailiac crests. The hip circumference was measured as the maximal circumference over the hip circumference (HC) at the level of the widest diameter around the gluteal protuberance in centimeters. Body mass index (BMI) was calculated by dividing weight (kg) by height squared (m^2^). The waist-to-hip ratio (WHR) was calculated by dividing the waist circumference (cm) by the hip circumference (cm). Waist-to-height ratio was calculated by dividing the waist circumference (cm) by the height circumference (cm). Other calculated adiposity indices were as follows:

(1) Conicity index (CI) [29]:
  (1) $$\begin{array}{c} \text{CI}=\frac{\text{Waist Circumference }\left(m\right)}{\left[0.109\times \sqrt{\text{Weight }\left(Kg\right)/\text{Height }\left(m\right)}\right]} \end{array}$$
(2) Abdominal volume index (AVI) [30]:
  (2) AVI=2Waist  C cm2+0.7Waist  C cm−Hip  C cm21000
(3) Body Adiposity Index (BAI) [31]:
  (3) $$\begin{array}{c} \text{BAI}=\frac{\text{Hip Circumference }\left(cm\right)}{{\left[\text{Height }\left(m\right)\right]}^{1.5}}-18. \end{array}$$

### 2.5. Biochemical Assays

Venous blood samples were collected after an overnight fast (12–16 hours) between 7 am and 10 am. About 5 mL of venous blood was drawn from the antecubital vein of which four mL was dispensed into vacutainer® plain tubes and one mL was dispensed into fluoride oxalate tubes. After centrifugation at 500 ×g for 15 minutes, the serum and plasma were stored at −80°C until assayed. Parameters that were determined include fasting blood glucose (FBG) to exclude participants with diabetes, urea, creatinine, uric acid, potassium, sodium, and chloride. The methods adopted for the determination of the urea, creatinine, and uric acid were predetermined by the reagent manufacturer (Dialab GmbH, IZ-NÖ Süd, Hondastrasse, A-2351 Wiener Neudorf, Austria). Ion Selective Electrode (ISE) method was used to assay electrolytes (AVL 9180 Electrolyte Analyzer, Roche Diagnostics, Switzerland, http://www.roche.com/index.htm) at the Komfo Anokye Teaching Hospital in Kumasi. All other biochemical analytical investigations were carried out at the Kumasi Centre for Collaborative Research in Tropical Medicine (KCCR) located at the Kwame Nkrumah University of Science and Technology, Kumasi.

### 2.6. Clinical Assessment

All the 241 participants underwent clinical assessment to determine target organ damage. Detailed history, physical examination, chest X-ray, 12-lead resting electrocardiogram (ECG), and transthoracic echocardiogram (ECHO) were done. All diagnoses and interpretation were performed by consultant radiologists and cardiologists.

### 2.7. Diagnosis of Chronic Kidney Disease

Estimate glomerular filtration rate (GFR) was calculated from serum creatinine according to Cockcroft-Gault CG equation as follows:

(CG) $$\begin{array}{c} eGFR=\frac{\left(140-Age\right)\times \text{Weight}}{72\times \text{Serum Creatinine}}\left(\;\times \;0.85\text{ if female}\;\right). \end{array}$$

### 2.8. Four-Variable Modification of Diet in Renal Disease

(4v-MDRD) eGFR=186×SCr−1.154×Age−0.203×1.212×0.742  if  female.

The eGFR results from the various renal function equations were used to stratify the study population into five categories corresponding to the five stages of CKD in the K/DOQI CKD classification [32]. Chronic kidney disease was classified as eGFR < 60 mL/min/1.73 m^2^ (stages 3, 4 and 5; see Table 5) [33].

### 2.9. Diagnosis of Heart Failure

Heart failure was diagnosed, using the modified Framingham criteria for diagnosis of heart failure [34]. Major criteria included paroxysmal nocturnal dyspnea, raised jugular venous pressure, cardiomegaly, basal crepitation, S3 gallop, and acute pulmonary oedema. Minor criteria included tachycardia, orthopnea, exertional dyspnea, nocturnal cough, hepatomegaly, and diuretic use. Heart failure was diagnosed if the participant had two major and one minor or one major and two minor criteria. Other complications of hypertension included cardiomegaly (without heart failure), stroke or transient ischaemic attack, and chronic kidney disease.

### 2.10. Statistical Analysis

Normality of all continuous variables was tested. All nonparametric variables were normalized by log transformation before analysis. Results obtained after analysis of log transformed variables were converted by antilog. Continuous variables were expressed as their mean ± SEM, whereas categorical variables were presented as count and proportion. Comparisons of the general characteristics of the hypertensive group against the normotensive group were performed using unpaired* t*-tests, chi square (*χ* ^2^) tests, or Fisher exact tests where appropriate. Analysis of variance (ANOVA) and Bonferroni post hoc test were used to compare more than two means, whilst a post hoc linear contrast test was used for trend analysis of continuous variables. A level of *p* < 0.05 was considered as statistically significant for all analyses. IBM Statistical Package for the Social Sciences (SPSS Inc., Chicago, USA (http://www.spss.com) version 20.00) and GraphPad Prism version 6.00 GraphPad software, San Diego, California, USA (http://www.graphpad.com/), for windows were used for statistical analysis where appropriate.

## 3. Results

Two hundred and forty-one (241) adult Ghanaians were recruited for the study. The sociodemographic characteristics of the participants, as shown in Table 2, involved 61 (25.3%) normotensives and 180 (74.7%) hypertensives, of which 40 patients representing 16.6% of the study population were newly diagnosed hypertensives who were not on any hypertensive drugs and were classified as drug-naive. Female participation was higher in each of the three categories of subjects. The informal sector employees dominated the case group. Significantly self-reported alcohol intake and smoking were both found to be more prevalent among the hypertensives, peaking in each case in the drug-naive group. No significant differences were recorded in the rest of the sociodemographic parameters assessed between the three different groups of participants evaluated in this study (see Table 2).

Among all the parameters evaluated as shown in Table 3, no statistically significant variation was recorded between the newly diagnosed hypertensives and their counterparts on treatment. Higher anthropometric indices were recorded among both hypertension groups compared with the normotensives; however, in the case of body mass index (BMI) and waist-to-hip ratio (WHR) the difference was not statistically significant. The hypertensives presented with significantly higher mean serum biochemical indices of renal dysfunction and electrolytes levels than their normotensive counterparts. The average concentration of microalbumin excreted in the urine as well as the glomerular filtration rate estimated by both 4v-MDRD and CG equations was comparable among the treatment-naïve hypertensives and the normotensives (see Table 3).

Among the general study population after adjusting for age and gender, positive additive changes in waist-to-height ratio (WHtR) as well as both the abdominal body frame factors (waist circumference (WC); hip circumference (HC)), the abdominal body region volumetric models for central obesity (conicity index (CI); abdominal volume index (AVI)) and body fat deposition (body adiposity index (BAI)) were associated with corresponding incremental changes in the haemodynamic measures (hypertension), the serum biochemical kidney profile measures, and renal insufficiency (decreasing eGFR). Among the hypertensive subpopulation, positive association was observed between the anthropometric measures and the serum biochemical kidney profile (K^+^, Na^+^, Cl^−^, urea, creatinine, and UA) as well as renal insufficiency. With the exception of pulse, no significant correlation was observed between the anthropometric measures and the haemodynamic measures among the hypertensive subpopulation.

Among both the general study population and the hypertension subgroup increasing levels of serum potassium, urea, creatinine, and uric acid were associated with increasing renal insufficiency. Significant association between the haemodynamic measures and the serum biochemical kidney profile was more profound in the general study population than the hypertension subgroup (see Table 4).

Among the total population of 241 participants, 13.3% presented with chronic kidney disease (CKD) when assessed by the four-variable modification of diet in renal disease 4v-MDRD equation. The Cockcroft-Gault CG recorded 16.6% and the chronic kidney disease epidemiology collaboration (CKD-EPI) equation recorded 14.5%. All patients presenting with CKD belong to the hypertension group. With the exception of one study participant who was observed to be in stage four by the Cockcroft-Gault CG equation, all CKD patients observed were in the third stage (see Table 5).

**Figure 1:**
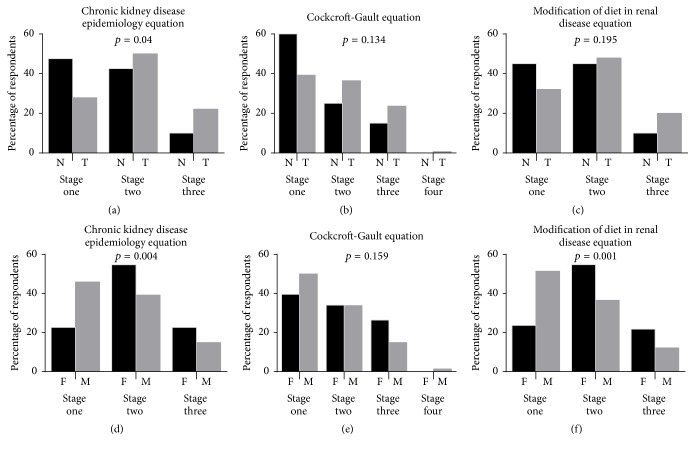
Estimates of the prevalence of CKD in the study population using the renal function equations stratified by therapy and gender. N: drug naïve, T: therapy, F: female, and M: male.

Among the hypertensive patients who were newly diagnosed and thus treatment-naïve and those who were on therapy irrespective of the equation used in assessing glomerular filtration rate, the group on therapy recorded a greater percentage of end-stage chronic kidney disease (CKD) than their naïve counterparts; however, with the exception of the chronic kidney disease epidemiology collaboration (CKD-EPI) equation (see Figure 1(a)), this difference was not statistically significant (see Figures 1(a) and 1(c)). With the exception of the Cockcroft-Gault CG equation (see Figure 1(e)), significant gender differences in the incidence of CKD were observed. Using the CKD-EPI equation, 22.6% of hypertensive females presented with CKD compared to 14.9% of hypertensive males (see Figure 1(d)). Twenty-one and seven percent (21.7%) of females compared to 12.2% of males presented with CKD as per the four-variable modification of diet in renal disease 4v-MDRD equation (see Figure 1(f)).

Irrespective of the equation used in estimating the glomerular filtration rate (GFR), self-reported nonalcoholics presented with significantly higher percentage of chronic kidney disease (CKD) than the alcoholics (23.6% versus 16.7%; 30.6% versus 16.7%; and 20.8% versus 15.7) for chronic kidney disease epidemiology collaboration (CKD-EPI), Cockcroft-Gault CG, and four-variable modification of diet in renal disease 4v-MDRD equations, respectively.

**Figure 2:**
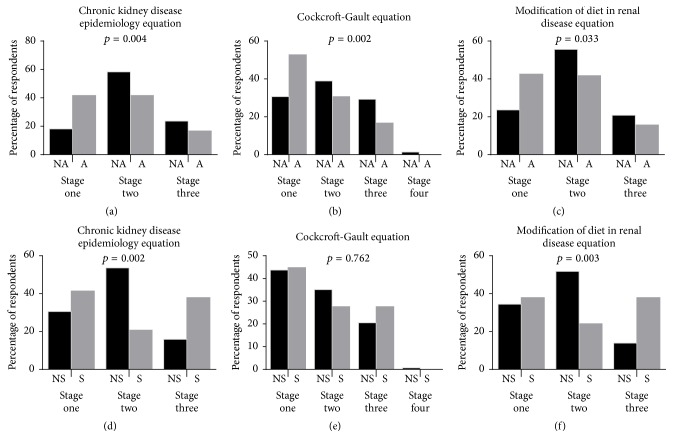
Estimates of the prevalence of CKD in the study population using the renal function equations stratified by alcohol consumption and smoking status. NA: nonalcoholic, A: alcoholic, S: smoker, and NS: nonsmoker.

From Figure 2(d), significantly higher percentage of smokers (37.9%) were observed with CKD, compared to their counterparts who did not smoke (15.9%), a similar outcome was observed with the use of 4v-MDRD where (37.9%) of smokers presented with CKD compared to (13.9%) of nonsmokers (see Figure 2).

**Figure 3:**
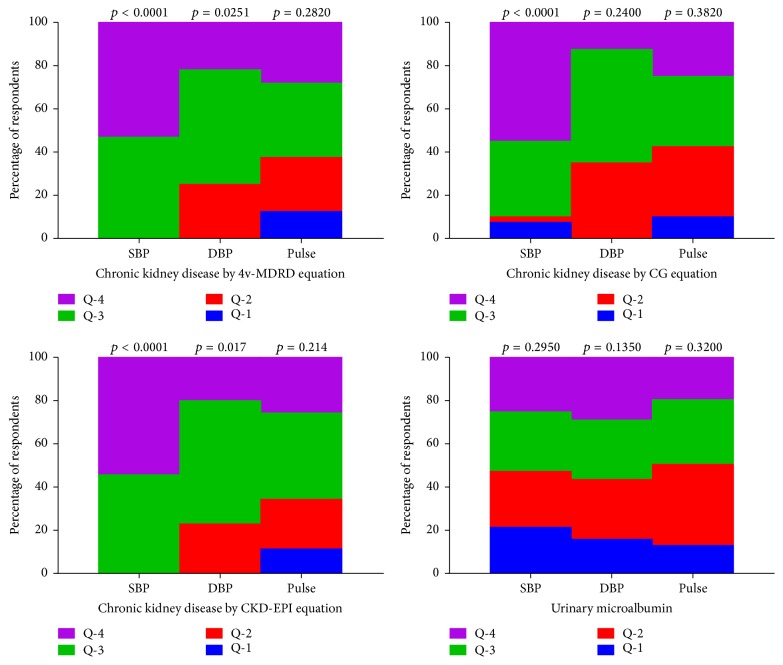
Renal insufficiency quartile cluster distributions with haemodynamic parameters. SBP: systolic blood pressure, DBP: diastolic blood pressure, Q-1: first quartile, Q-2: second quartile, Q-3: third quartile, and Q-4: fourth quartile. *p* value for linear by linear association is significant at 0.05 for two tail.

Almost all participants presenting with chronic kidney disease (CKD) clustered at the upper quartiles (3rd and 4th) of systolic blood pressure. The exception included 10% of CKD subjects who clustered at the lower quartiles (7.5% and 2.5% for 1st and 2nd quartiles, resp.) of systolic blood pressure when Cockcroft-Gault CG equation was used to estimate the glomerular filtration rate. Percentage cluster distribution by CKD was observed to be significantly tilted toward the upper quartiles of diastolic blood pressure. Though not statistically significant, the majority of CKD participants clustered in the third and fourth quartiles of pulse, irrespective of the renal function equation used. The majority of participants presenting with microalbuminuria clustered in the upper quartiles of the haemodynamic parameters evaluated (see Figure 3).

**Figure 4:**
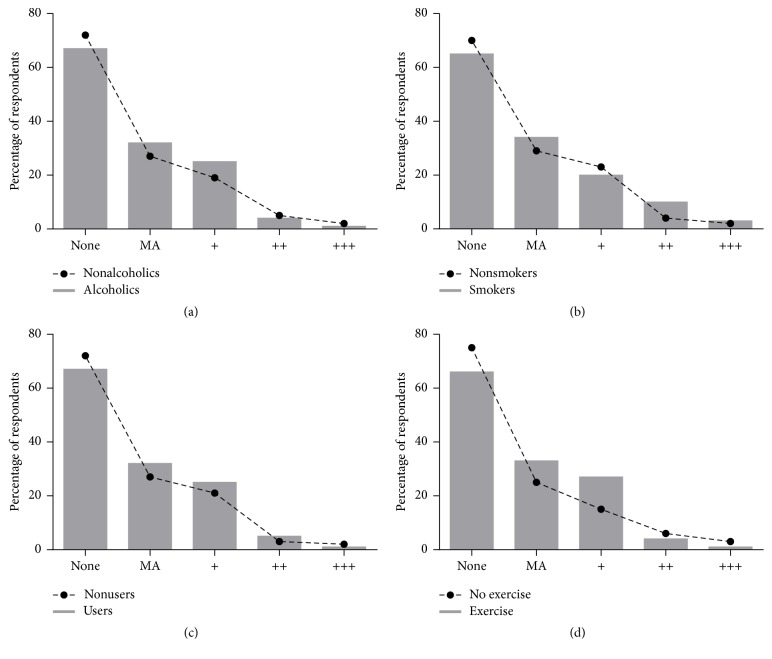
Prevalence of microalbuminuria among hypertension subjects stratified by sociodemographic characteristics. MA: microalbuminuria. (a) Alcohol intake, (b) smoking status, (c) herbal medicine intake, and (d) physical activity level.

The prevalence of microalbuminuria among the alcoholic subjects was 32.4% compared to 27.8% among the nonalcoholics. Study participants who smoked recorded a higher percentage of microalbuminuria compared to nonsmokers and also a higher number of smokers were found to cluster at increasing levels of microalbuminuria compared to nonsmokers. Patients using herbal medicine had higher microalbuminuria (32.7%) than those who were not using herbal medications (27.6%). Even though higher microalbuminuria was observed among the physically active group, severity of microalbuminuria was more profound in their nonactive counterparts (see Figure 4).

In general, significant additive linear relationship was observed for all serum renal biochemical parameters assayed with progressive quartile increment of haemodynamic indices. The exception was observed with the average levels of uric acid among the diastolic quartile cluster distribution, where no significant linearity was observed.

Irrespective of the renal function equation used in the estimation of the glomerular filtration rate among the study population, participants presenting with cardiac abnormalities (left ventricular hypertrophy-electrocardiograph, left ventricular hypertrophy-echocardiograph, and cardiomegaly-X-ray) recorded significant prevalence of chronic kidney disease compared to their counterparts who were without the specific abnormal heart condition (see Table 6).

## 4. Discussion

Earlier reports by Addo et al. [35] and Osafo et al. [36] put the calculated incidence of renal insufficiency (eGFR < 60 mL/min/1.73 m^2^; stages 3, 4, and 5) among different hypertensive populations in Accra Ghana at 4.1% and 27.8%, respectively. The estimates of hypertensive kidney target organ damage burden for the present study (17.80%–22.22%) compared to the earlier records in the previous studies but were higher than the former and lower than the latter. The difference in CKD burden for the hypertensive cohort was perhaps due to differences in study population characteristics; thus, the work of Addo et al. [35] involved a population survey whilst that of Osafo et al. [36] and the present study were hospital-based studies. Again the fact that the study of Osafo et al. [36] included 14.7% of subjects with preexisting diabetes mellitus whereas the present study involved only nondiabetic hypertensives may account for the higher CKD incidence recorded in the previous report. Adu [37] in an editorial in the Ghana Medical Journal estimated the prevalence of hypertension in the African population at 10% and posited that hypertension accounts for 38.8% of the aetiology of CKD and is possibly the leading cause of target kidney organ damage among this population.

The incidence of CKD was higher among the hypertensive study participants on treatment than the treatment-naïve group. The increased duration of chronic diseases like hypertension has usually been linked with deterioration of sequelae and survival. Also the institution of drug therapy in the management of hypertension is usually associated with advancing disease state and this may account for the higher target kidney organ damage observed among the hypertensives on treatment.

Gender differences in health vary according to differential vulnerabilities in males and females, which may be as a result of biological or sociodemographic differences [38, 39]. In the case of chronic kidney disease among hypertensives, vulnerability seems to be more tilted toward the female gender than the male gender [4, 40]. This finding is in agreement with observations made in this study (see Figure 1). Wang et al. [40] have suggested that the stronger association between obesity and chronic kidney disease in women than in men could account for this phenomenon. In Ghana, obesity is reported to be significantly higher among females than in males [13, 41]. Among hypertensives, obesity was found to be significantly higher (*p* = 0.0337) among females (57.5%) compared to the males (40.5%).

The raging debate of alcohol consumption as a modifiable lifestyle trait influencing both hypertension and kidney outcomes in the scientific literature has become even more intense in recent times because of the great importance it confers due to the seemingly large numbers of the population any prognostic outcome may affect. In the current study, self-reported alcohol consumption was recorded among more than half of the study population (130 (53.9%)) with a significantly higher presence among the hypertensives than the normotensives (see Table 1). Deleterious association of alcohol consumption with hypertension is available in the Ghanaian literature [10, 42, 43]. Plausible mechanisms elucidated for such adverse effects include stimulation of corticosteroid, catecholamine, vasopressin production, and drug interaction causing resistance to drug therapy and direct pressor effect due to alcohol induced arteriolar vasoconstriction [44–46].

The findings of the current study are consistent with those of Renaud et al. [47] and Reynolds et al. [48] both of whom reported an inverse association between alcohol consumption and chronic kidney disease among hypertensives (see Figure 2). However, due to our inability to determine the threshold dose level of alcohol intake among the study population, drawing inferences for causality of alcohol dose response was not possible [46, 49]. It is therefore appropriate to concur with Reynolds et al. [48] who called for caution in the interpretation of similar results, since epidemiologically a suggested J- or U-shaped relationship exists between alcohol consumption and coronary heart diseases. Thus, the apparent benefits of moderate drinking on kidney and coronary heart disease mortality are offset at higher drinking levels by increasing risk of death from other types of heart diseases (cardiomyopathy, arrhythmia, etc.), neurological disorders, cancer, liver cirrhosis, and traffic accidents [46, 50].

Despite the recent report from the HUNT study suggesting smoking is not a major determinant of adverse blood pressure and other component cardiovascular disease risk factors [51] and the report from the population-based Malmö-Diet-and-Cancer-Cohort which found that the associated risk of cardiovascular disease and cardiovascular disease mortality conferred by genetic variation on chromosome 9p21 may be attenuated by smoking [52], there is consensus on the association of smoking with adverse cardiovascular and renal outcome [53–59].

In the present study, self-reported smoking was 13.3% among the total study population and significantly higher among the hypertensives compared to normotensives (16.1% versus 4.9%). In agreement with the findings of adverse renal events associated with cigarette smoking among various study populations [53, 60–63], current smoking status was associated with chronic kidney disease among hypertensives (see Figure 2). Plausible mechanisms of smoking attributed renal injury include increased blood pressure and heart rate; altered diurnal blood pressure rhythm; increased sympathetic nerve activity; nicotine induced mesangial cell proliferation and increased production of fibronectin; arteriosclerosis of renal and intrarenal arteries and arterioles; growth factor activation (angiotensin II, endothelin-1, and TGF-1); oxidative stress; impaired lipoprotein and glycosaminoglycan metabolism; tubulotoxicity; direct endothelial cell toxicity; increased aggregation of platelet; modulation of immune mechanisms; vasopressin-mediated antidiuresis and insulin resistance [4, 64].

Excess weight gain is a major risk factor for essential hypertension and end-stage renal disease (ESRD) [65, 66]. In the present study, higher anthropometric indices were recorded among the hypertensive groups compared to the normotensives and, after adjusting for age and gender, positive additive changes in anthropometric indices were associated with corresponding incremental changes in the haemodynamic measurements (hypertension), the serum kidney profile estimates, and renal insufficiency (decreasing eGFR). According to the Guyton hypothesis, sustained hypertension can occur only when there is a derangement of pressure natriuresis [6, 67, 68]. Obesity related resetting of the kidney-fluid apparatus to a hypertensive level is consistent with the model of hypertension by volume overload [69]. Documented pathogenic mechanisms include enhanced sympathetic nervous system activity, increased activity of the systemic renin-angiotensin system, low atrial natriuretic peptide levels, and physical compression of the kidneys by fat deposits within and around the kidneys, coupled with increased abdominal pressure due to accumulation of excess visceral fat, all of which can cause sodium retention [65, 70–72]. This enhanced sodium avidity shifts the pressure natriuresis curve to the right, thereby necessitating higher arterial pressure to excrete the day's salt intake and maintain sodium balance and volume homeostasis [67, 73].

There is an accumulating body of clinical and experimental data implicating obesity as an important causative factor in renal disease [74–76]. Several alterations in renal structure and function have been associated with obesity [77]. The compensatory mechanisms to maintain sodium balance in obesity in the long term lead to increased systemic arterial pressure, creating a haemodynamic burden on the kidneys which causes glomerular injury [65]. Deposition of extracellular matrix throughout the renal medulla and the ducts of Bellini compresses the renal parenchyma toward the pole of the kidney resulting in the formation of round-shaped, enlarged kidneys in obese subjects [69].

There is intense debate as to whether hypertension causes kidney disease or vice versa [4]. High blood pressure is a key pathogenic factor that contributes to the deterioration of kidney function [78]. Conversely, chronic kidney disease (CKD) is the most common form of secondary hypertension [6], thus making the kidney both a cause and a victim of hypertension [5]. In the current study, chronic kidney disease was observed only among the participants presenting with hypertension, irrespective of the predictive equation used in estimating the glomerular filtration rate. The association between hypertension and CKD was also evident as percentage cluster distribution of participants presenting with chronic kidney disease predominantly segregated with the upper quartiles (3rd and 4th quartiles) of the haemodynamic indices (SBP, DBP, and pulse; see Figure 3). The strength of association was found to be more pronounced with systolic blood pressure rather than diastolic blood pressure, posing a greater risk for cardiovascular events and kidney disease progression.

Serum elevation of kidney excretory products like electrolytes and metabolites of purine and amino acid catabolism (uric acid and urea) in hypertensives has traditionally been attributed to decreased glomerular filtration rate (GFR) as a result of the effect of hypertension on renal function. A reduction in renal blood flow as a consequence of increasing renal vascular resistance leads to decreased distal tubular flow rate which leads to increased reabsorption and decreased excretion [68, 79, 80]. Significantly higher levels of serum electrolytes as well as urea and uric acid were observed among the hypertensive subgroup compared to their normotensive counterparts (see Table 2). The assertion that serum kidney excretory products are only secondary to residual confounding factors that have a pathogenically inert relationship with hypertension and renal disease is giving way. Serum kidney excretory products are now viewed as pathophysiologically active independent attribution factors for the cause and progression of hypertension and renal disease [81–85].

Linear additive relationship was observed for all serum electrolytes as well as urea and uric acid with progressive quartile increment of haemodynamic indices measured in this study. After adjusting for age and gender in the present study, surfeit urea and uric acid were significantly associated with renal insufficiency (see Table 3). Often considered a beneficial antioxidant, recent epidemiologic and experimental studies have demonstrated that hyperuricaemia is a major and an independent risk factor for the development of renal disease, hypertension, and adverse cardiovascular outcome [79, 83, 84, 86, 87]. Possible adverse effects of uric acid on the vasculature have been linked to increased chemokine and cytokine expression [88], induction of renal vasoconstriction mediated by endothelial dysfunction, activation of the renin-angiotensin system [87], and stimulation of oxidative stress in vascular smooth muscle cell (VSMC) proliferation, mediated by the mitogen-activated protein (MAP) kinase pathway [89] and increased vascular C-reactive protein (CRP) expression [88].

## 5. Conclusion

In this urban population, chronic kidney disease was associated with hypertension and cardiac abnormalities. Adiposity was associated with hypertension and renal insufficiency. Modifiable lifestyle practices (smoking and alcoholism) increased the risk of hypertension, whilst smoking increased kidney target organ damage among hypertensives.

## Figures and Tables

**Table 1:** Equations for chronic kidney disease epidemiology collaboration (CKD-EPI).

Gender	Serum creatinine *µ*mol/L (mg/dL)	Estimated glomerular equation
Female	≤62 (≤0.7)	$eGFR=166\times \left(\frac{\text{Serum Creatinine}}{0.7^{-0.329}}\right)\times {\left(0.993\right)}^{Age}$
Female	>62 (>0.7)	$eGFR=166\times \left(\frac{\text{Serum Creatinine}}{0.7^{-1.209}}\right)\times {\left(0.993\right)}^{Age}$
Male	≤80 (≤0.9)	$eGFR=163\times \left(\frac{\text{Serum Creatinine}}{0.9^{-0.411}}\right)\times {\left(0.993\right)}^{Age}$
Male	>80 (>0.9)	$eGFR=163\times \left(\frac{\text{Serum Creatinine}}{0.9^{-1.209}}\right)\times {\left(0.993\right)}^{Age}$

**Table 2:** Sociodemographic characteristics of the population under study stratified by hypertension and treatment status.

Parameter	Control	HPT-naïve	HPT-therapy	*p* value
Respondents	61 (25.3)	40 (16.6)	140 (58.1)	*nd*
Female	45 (73.8)	22 (55.0)	84 (60.0)	0.0980
Male	16 (26.2)	18 (45.0)	56 (40.0)	
Formal sector	45 (74.2)	10 (24.4)	44 (31.7)	<0.0001
Informal sector	16 (25.8)	30 (75.6)	96 (68.3)	
Alcoholics	22 (36.1)	32 (80.0)	76 (54.3)	<0.0001
Smokers	3 (4.9)	9 (22.5)	20 (14.3)	0.0340
Exercise	36 (59.0)	27 (67.5)	89 (63.6)	0.6760
Positive family history	40 (65.6)	31 (77.5)	104 (74.3)	0.3330
Herbal medicine intake	33 (54.1)	24 (60.0)	79 (56.4)	0.8430
Nonprescribed drug intake	36 (59.0)	22 (55.0)	82 (58.6)	0.9090

Data is presented as figure with corresponding percentage in parenthesis. HPT-naïve: newly diagnosed hypertensive not on antihypertensive drugs; HPT-therapy: hypertensives on antihypertensives. *p* is significant at 0.005.

**Table 3:** Haemodynamic, anthropometric, serum biochemical kidney profile and estimated glomerular filtration rate of the study population stratified by hypertension status.

Parameter	Control	HPT-naïve	HPT-therapy	*p* value
SBP (mmHg)	117.38 ± 0.96	152.00 ± 3.27	155.46 ± 1.82	<0.0001
DBP (mmHg)	73.28 ± 0.77	100.50 ± 1.34	101.46 ± 0.94	<0.0001
Pulse (BPM)	51.41 ± 0.91	76.85 ± 2.20	81.74 ± 1.77	<0.0001
MAP (mmHg)	87.98 ± 0.63	117.67 ± 1.81	119.46 ± 1.01	<0.0001
WC (cm)	69.10 ± 1.57	97.65 ± 2.80	95.69 ± 1.58	<0.0001
HC (cm)	71.82 ± 1.56	102.20 ± 3.20	101.42 ± 2.12	<0.0001
BMI (Kg/m^2^)	29.36 ± 0.65	29.80 ± 0.71^a^	29.52 ± 0.39^a^	0.9018
WHR	0.96 ± 0.01	0.97 ± 0.01^a^	0.97 ± 0.0^a^	0.9356
WHtR	0.45 ± 0.01	0.60 ± 0.11	0.59 ± 0.13	<0.0001
AVI	9.86 ± 0.45	19.74 ± 1.16	19.09 ± 0.66	<0.0001
Conicity index	0.929 ± 0.11	1.28 ± 0.04	1.25 ± 0.02	<0.0001
BAI	19.49 ± 0.90	31.14 ± 1.60	31.40 ± 1.17	<0.0001
Potassium (mmol/L)	3.20 ± 1.03	3.76 ± 1.05	3.82 ± 1.03	0.0001
Sodium (mmol/L)	134.89 ± 1.35	139.43 ± 3.20	146.86 ± 2.39	<0.0001
Chloride (mmol/L)	94.89 ± 1.35	106.13 ± 1.12	112.73 ± 1.03	<0.0001
Urea (mmol/L)	3.71 ± 0.09	5.66 ± 0.31	5.77 ± 0.14	<0.0001
Creatinine (*µ*mol/L)	69.08 ± 1.48	86.81 ± 4.29	91.71 ± 2.38	<0.0001
Uric acid (*µ*mol/L)	125.68 ± 1.02	157.19 ± 1.07	176.66 ± 1.05	<0.0001
Microalbuminuria (mg)	4.52 ± 1.14	5.60 ± 1.21^a^	7.38 ± 1.12	0.0336
4v-MDRD (mL/min/1.73 m^2^)	127.63 ± 1.03	101.78 ± 1.13^a^	87.05 ± 1.06	0.0002
CG (mL/min/1.73 m^2^)	130.24 ± 1.03	103.26 ± 1.12^a^	82.61 ± 1.05	<0.0001
CKD-EPI (mL/min/1.73 m^2^)	131.23 ± 1.03	90.33 ± 1.06	81.49 ± 1.05	<0.0001

Data is presented as mean ± standard error of the mean. HPT-naïve: newly diagnosed hypertensive not on antihypertensive drugs, HPT-therapy: hypertensives on antihypertensives, SBP: systolic blood pressure, DBP: diastolic blood pressure, MAP: mean arterial pressure, WC: waist circumference, HC: hip circumference, BMI: body mass index, WHR: waist-to-hip ratio, WHtR: waist-to-height ratio, AVI: abdominal volume index, BAI: body adiposity index, 4v-MDRD: four-variable modification of diet in renal disease, CG: Cockcroft-Gault, CKD-EPI: chronic kidney disease epidemiology collaboration. Bonferroni post hoc^a^: no statistically significant difference compared with control.

**Table 4:** Partial correlation coefficients of haemodynamic parameters, anthropometric variables, and biochemical kidney markers of study population (upper right sided) and indices of hypertension group (lower left sided), adjusted for age and gender.

Index	SBP	DBP	Pulse	MAP	WC	HC	BMI	WHR	WHtR	CI	AVI	BAI	K^+^	Na^+^	CL^−^	Urea	Creat	UA	MA	MDRD	CG	EPI
SBP		.51^*∗∗*^	.20^*∗∗*^	.85^*∗∗*^	.20^*∗∗*^	.16^*∗*^	−0.03	0.04	.19^*∗∗*^	.24^*∗∗*^	.18^*∗∗*^	.30^*∗∗*^	0.07	.23^*∗∗*^	.39^*∗∗*^	.21^*∗∗*^	.15^*∗*^	0.10	−0.09	−0.03	−0.01	−0.06
DBP	.39^*∗∗*^		.25^*∗∗*^	.89^*∗∗*^	.19^*∗∗*^	.19^*∗∗*^	0.05	0.00	0.12	.17^*∗∗*^	.16^*∗*^	.28^*∗∗*^	0.01	.35^*∗∗*^	.52^*∗∗*^	0.11	.13^*∗*^	0.01	0.01	0.00	0.08	−0.02
Pulse	0.02	0.01		.26^*∗∗*^	.64^*∗∗*^	.69^*∗∗*^	.17^*∗∗*^	0.07	.58^*∗∗*^	.57^*∗∗*^	.68^*∗∗*^	.66^*∗∗*^	.22^*∗∗*^	.20^*∗∗*^	.40^*∗∗*^	.22^*∗∗*^	0.05	.29^*∗∗*^	0.01	−0.01	0.11	−0.01
MAP	.83^*∗∗*^	.83^*∗∗*^	0.02		.22^*∗∗*^	.20^*∗∗*^	0.01	0.02	.18^*∗∗*^	.24^*∗∗*^	.19^*∗∗*^	.31^*∗∗*^	0.04	.33^*∗∗*^	.53^*∗∗*^	.18^*∗∗*^	.16^*∗*^	0.06	−0.04	−0.01	0.04	−0.05
WC	0.04	−0.07	.60^*∗∗*^	−0.02		.95^*∗∗*^	.37^*∗∗*^	0.1	.93^*∗∗*^	.89^*∗∗*^	.99^*∗∗*^	.86^*∗∗*^	.19^*∗∗*^	.14^*∗*^	.40^*∗∗*^	.29^*∗∗*^	.21^*∗∗*^	.44^*∗∗*^	0.08	−.18^*∗∗*^	0.03	−.20^*∗∗*^
HC	0.02	−0.04	.66^*∗∗*^	−0.01	.94^*∗∗*^		.33^*∗∗*^	−0.12	.88^*∗∗*^	.84^*∗∗*^	.96^*∗∗*^	.90^*∗∗*^	.23^*∗∗*^	.13^*∗*^	.35^*∗∗*^	.26^*∗∗*^	.21^*∗∗*^	.47^*∗∗*^	0.06	−.21^*∗∗*^	−0.01	−.21^*∗∗*^
BMI	−0.06	0.01	.18^*∗*^	−0.03	.37^*∗∗*^	.32^*∗∗*^		0.03	.47^*∗∗*^	−0.03	.33^*∗∗*^	.43^*∗∗*^	0.02	0.00	0.09	0.01	0.06	−0.03	0.12	−0.11	.19^*∗∗*^	−0.10
WHR	0.04	−0.02	0.08	0.01	0.07	−.16^*∗*^	0.00		0.10	0.10	0.09	−0.10	−0.04	0.00	0.03	0.07	−0.08	−0.09	0.01	.17^*∗∗*^	.18^*∗∗*^	0.09
WHtR	0.07	−0.09	.55^*∗∗*^	−0.01	.93^*∗∗*^	.86^*∗∗*^	.42^*∗∗*^	0.07		.80^*∗∗*^	.92^*∗∗*^	.95^*∗∗*^	.16^*∗*^	0.12	.33^*∗∗*^	.29^*∗∗*^	.20^*∗∗*^	.43^*∗∗*^	0.1	−.21^*∗∗*^	−0.04	−.22^*∗∗*^
CI	0.10	−0.08	.51^*∗∗*^	0.01	.87^*∗∗*^	.81^*∗∗*^	−0.09	0.08	.80^*∗∗*^		.89^*∗∗*^	.75^*∗∗*^	.16^*∗*^	.15^*∗*^	.37^*∗∗*^	.29^*∗∗*^	.19^*∗∗*^	.49^*∗∗*^	0.05	−.16^*∗*^	−0.1	−.18^*∗∗*^
AVI	0.04	−0.07	.65^*∗∗*^	−0.02	.99^*∗∗*^	.96^*∗∗*^	.33^*∗∗*^	0.07	.92^*∗∗*^	.87^*∗∗*^		.88^*∗∗*^	.21^*∗∗*^	0.11	.36^*∗∗*^	.29^*∗∗*^	.20^*∗∗*^	.46^*∗∗*^	0.07	−.18^*∗∗*^	0.01	−.20^*∗∗*^
BAI	0.05	−0.07	.60^*∗∗*^	−0.01	.86^*∗∗*^	.89^*∗∗*^	.39^*∗∗*^	−0.13	.95^*∗∗*^	.73^*∗∗*^	.87^*∗∗*^		.24^*∗∗*^	.25^*∗∗*^	.40^*∗∗*^	.35^*∗∗*^	.27^*∗∗*^	.52^*∗∗*^	0.11	−.30^*∗∗*^	−.14^*∗*^	−.32^*∗∗*^
K^+^	0.02	−0.10	.21^*∗∗*^	−0.05	.20^*∗∗*^	.24^*∗∗*^	0.09	−0.05	.19^*∗*^	.15^*∗*^	.22^*∗∗*^	.21^*∗∗*^		.21^*∗∗*^	0.11	.20^*∗∗*^	.16^*∗*^	.19^*∗∗*^	−0.03	−.14^*∗*^	−0.1	−0.11
Na^+^	0.06	.16^*∗*^	0.04	0.14	−0.06	−0.04	−0.04	−0.02	−0.04	0.04^*∗*^	0.06	−0.02	.20^*∗∗*^		.35^*∗∗*^	.16^*∗*^	0.10	−0.03	−0.02	0.08	0.11	0.02
CL^−^	0.12	0.10	0.09	0.13	0.14	0.11	0.04	0.06	0.10	0.12	.16^*∗*^	0.06	0.03	0.03		.31^*∗∗*^	.18^*∗∗*^	.21^*∗∗*^	0.08	−0.04	0.08	−0.08
Urea	0.09	−0.12	0.08	−0.02	.20^*∗∗*^	.17^*∗*^	0.00	0.09	.22^*∗∗*^	.19^*∗*^	.21^*∗∗*^	.20^*∗∗*^	.18^*∗*^	0.04	0.02		.38^*∗∗*^	.29^*∗∗*^	−0.05	−.23^*∗∗*^	−.18^*∗∗*^	−.27^*∗∗*^
Creat	0.09	0.04	−0.03	0.08	.17^*∗*^	.17^*∗*^	0.07	−0.11	.17^*∗*^	0.14^*∗*^	.16^*∗*^	.17^*∗*^	0.15	0.05	0.06	.36^*∗∗*^		.30^*∗∗*^	0.02	−.80^*∗∗*^	−.73^*∗∗*^	−.79^*∗∗*^
UA	0.02	−0.14	.24^*∗∗*^	−0.07	.45^*∗∗*^	.46^*∗∗*^	−0.06	−0.13	.43^*∗∗*^	.49^*∗∗*^	.45^*∗∗*^	.42^*∗∗*^	.18^*∗*^	−0.12	0.11	.24^*∗∗*^	.27^*∗∗*^		0.02	−.29^*∗∗*^	−.27^*∗∗*^	−.27^*∗∗*^
MA	−0.13	−0.06	−0.04	−0.11	0.02	0.00	0.10	0.04	0.05	0.00	0.02	0.03	−0.04	−0.08	0.09	−0.09	0.03	0.03		−0.05	−0.03	−0.05
MDRD	−0.02	0.04	0.01	0.01	−.20^*∗∗*^	−.22^*∗∗*^	−0.13	0.21	−.22^*∗∗*^	−.16^*∗*^	−.19^*∗*^	−.24^*∗∗*^	−.15^*∗*^	0.10	0.00	−.23^*∗∗*^	−.81^*∗∗*^	−.29^*∗∗*^	−0.06		.92^*∗∗*^	.90^*∗∗*^
CG	−0.05	0.05	0.08	−0.01	−0.06	−0.09	.15^*∗*^	0.20	−0.12	−.19^*∗*^	−0.06	−.16^*∗*^	−0.12	0.07	0.04	−.23^*∗∗*^	−.77^*∗∗*^	−.29^*∗∗*^	−0.07	.94^*∗∗*^		.81^*∗∗*^
EPI	−0.03	0.05	0.04	0.01	−.20^*∗∗*^	−.21^*∗∗*^	−0.13	0.11	−.22^*∗∗*^	−.17^*∗*^	−.19^*∗*^	−.22^*∗∗*^	−0.12	0.05	−0.01	−.26^*∗∗*^	−.80^*∗∗*^	−.26^*∗∗*^	−0.06	.90^*∗∗*^	.83^*∗∗*^	

SBP: systolic blood pressure, DBP: diastolic blood pressure, MAP: mean arterial pressure, WC: waist circumference, HC: hip circumference, BMI: body mass index, WHR: waist-to-hip ratio, WHtR: waist-to-height ratio, CI: conicity index, AVI: abdominal volume index, K^+^: potassium, Na^+^: sodium, CL^−^: chloride, creat: creatinine, UA: uric acid, MA: microalbumin, MDRD: four-variable modification of diet in renal disease, CG: Cockcroft-Gault, and EPI: chronic kidney disease epidemiology collaboration. ^*∗*^Correlation is significant at 0.05 and ^*∗∗*^correlation is significant at 0.01.

**Table 5:** Estimates of the prevalence of CKD in the study population using the renal function equations.

Parameter	4v-MDRD			CG			CKD-EPI		

eGFR (mL/min/1.73 m^2^)	Total	Control	Case	Total	Control	Case	Total	Control	Case

Stage 1 (≥90)	120 (49.80)	57 (93.40)	63 (35.00)	136 (56.40)	57 (93.40)	79 (43.90)	117 (48.50)	59 (96.70)	58 (32.20)
Stage 2 (60 to 89)	89 (36.90)	4 (6.60)	85 (47.20)	65 (27.00)	4 (6.60)	61 (33.90)	89 (36.90)	2 (3.30)	87 (48.30)
Stage 3 (30 to 59)	32 (13.30)	0 (0.00)	32 (17.80)	39 (16.20)	0 (0.00)	39 (21.70)	35 (14.50)	0 (0.00)	35 (14.50)
Stage 4 (15 to 29)	0 (0.00)	0 (0.00)	0 (0.00)	1 (0.40)	0 (0.00)	1 (0.60)	0 (0.00)	0 (0.00)	0 (0.00)
Stage 5 (<15)	0 (0.00)	0 (0.00)	0 (0.00)	0 (0.00)	0 (0.00)	0 (0.00)	0 (0.00)	0 (0.00)	0 (0.00)

CKD (Stages 3 + 4 + 5)	32 (13.30)	0 (0.00)	32 (17.80)	40 (16.60)	0 (0.00)	40 (22.22)	35 (14.50)	0 (0.00)	35 (19.40)

Data is presented as absolute values with corresponding percentage in parenthesis. 4v-MDRD: four variable modification of diet in renal disease, CG: Cockcroft-Gault, CKD-EPI: chronic kidney disease epidemiology collaboration, eGFR: estimated glomerular filtration rate, and CKD: chronic kidney disease.

**Table 6:** Staged renal insufficiency stratified by the presence of clinical manifestation of cardiac disorder (nonspecific sinuses, left ventricular hypertrophy, and heart enlargement).

Parameter	Equation	Condition	Stage 1	Stage 2	Stage 3	Stage 4	*p* value
Left ventricular hypertrophy (electrocardiograph)	4v-MDRD	*Absent*	66 (74.12)	17 (19.10)	6 (6.74)	0 (0.00)	*p* < 0.0001
		*Present*	54 (35.53)	72 (47.37)	26 (17.11)	0 (0.00)	
	CG	*Absent*	68 (76.40)	12 (13.48)	9 (10.11)	0 (0.00)	*p* < 0.0001
		*Present*	68 (44.74)	53 (34.87)	30 (19.74)	1 (0.67)	
	CKD-EPI	*Absent*	66 (74.16)	17 (19.10)	6 (6.74)	0 (0.00)	*p* < 0.0001
		*Present*	51 (33.55)	72 (47.37)	29 (19.08)	0 (0.00)	

Left ventricular hypertrophy (echocardiograph)	4v-MDRD	*Absent*	62 (76.54)	16 (19.75)	3 (3.70)	0 (0.00)	*p* < 0.0001
		*Present*	58 (36.25)	73 (45.63)	29 (18.13)	0 (0.00)	
	CG	*Absent*	65 (80.25)	12 (14.83)	4 (4.94)	0 (0.00)	*p* < 0.0001
		*Present*	71 (44.38)	53 (33.13)	35 (21.88)	1 (0.63)	
	CKD-EPI	*Absent*	64 (79.00)	14 (17.30)	3 (3.70)	0 (0.00)	*p* < 0.0001
		*Present*	53 (33.10)	75 (46.90)	32 (20.00)	0 (0.00)	

Cardiomegaly (X-ray)	4v-MDRD	*Absent*	74 (71.80)	21 (20.40)	8 (7.80)	0 (0.00)	*p* < 0.0001
		*Present*	46 (33.30)	68 (49.30)	24 (17.40)	0 (0.00)	
	CG	*Absent*	79 (76.70)	18 (17.48)	6 (5.83)	0 (0.00)	*p* < 0.0001
		*Present*	57 (41.30)	47 (34.06)	33 (23.91)	1 (0.73)	
	CKD-EPI	*Absent*	64 (79.00)	14 (17.30)	3 (3.70)	0 (0.00)	*p* < 0.0001
		*Present*	53 (33.10)	75 (46.90)	32 (20.00)	0 (0.00)	

Data is presented as figure with percentage in parenthesis. 4v-MDRD: four-variable modification of diet in renal disease, CG: Cockcroft-Gault, and CKD-EPI: chronic kidney disease epidemiology collaboration equation.

## Additional Points

*Limitation of Study*. The threshold dose level of alcohol intake could not be established from this study; thus, inferences for causality of alcohol dose response were not feasible. Further studies will be required to help determine the threshold dose of alcohol intake.
